# Skin Hydration Monitoring Using a Microwave Sensor: Design, Fabrication, and In Vivo Analysis

**DOI:** 10.3390/s25113445

**Published:** 2025-05-30

**Authors:** Shabbir Chowdhury, Amir Ebrahimi, Kamran Ghorbani, Francisco Tovar-Lopez

**Affiliations:** School of Engineering, RMIT University, Melbourne, VIC 3001, Australia; s3779477@student.rmit.edu.au (S.C.); kamran.ghorbani@gmail.com (K.G.); francisco.tovarlopez@rmit.edu.au (F.T.-L.)

**Keywords:** hydration, radio frequency sensor, reflective sensors, skin hydration

## Abstract

This article introduces a microwave sensor tailored for skin hydration monitoring. The design enables wireless operation by separating the sensing component from the reader, making it ideal for wearable devices like wristbands. The sensor consists of a semi-lumped LC resonator coupled to an inductive coil reader, where the capacitive part of the sensing tag is in contact with the skin. The variations in the skin hydration level alter the dielectric properties of the skin, which, in turn, modify the resonances of the LC resonator. Experimental in vivo measurements confirmed the sensor’s ability to distinguish between four hydration conditions: wet skin, skin treated with moisturizer, untreated dry skin, and skin treated with Vaseline, by measuring the resonance frequencies of the sensor. Measurement of the input reflection coefficient ($S_{11}$) using a vector network analyzer (VNA) revealed distinct reflection poles and zeros for each condition, demonstrating the sensor’s effectiveness in detecting skin hydration levels. The sensing principle was analyzed using an equivalent circuit model and validated through measurements of a fabricated sensor prototype. The results confirm in vivo skin hydration monitoring by detecting frequency shifts in the reflection response within the 50–200 MHz range. The measurements and data analysis show less than 0.037% error in transmission zero ($f_{z}$) together with less than 1.5% error in transmission pole ($f_{p}$) while being used to detect skin hydration status on individual human subjects. The simplicity of the detection method, focusing on key frequency shifts, underscores the sensor’s potential as a practical and cost-effective solution for non-invasive skin hydration monitoring. This advancement holds significant potential for skincare and biomedical applications, enabling detection without complex signal processing.

## 1. Introduction

The health and functionality of human skin are greatly influenced by its hydration level, particularly in the outer layers. Healthy skin tissue typically contains approximately 10% water [1]. The actual water content varies depending on a person’s health, age, gender, and other physiological factors. Insufficient hydration can lead to significant issues, especially for children and the elderly [2]. Skin dehydration occurs when the skin loses more water than it receives, leading to dryness and reduced elasticity [3]. This can be due to various factors such as environmental conditions, prolonged sun exposure, or inadequate water intake. Dehydrated skin often feels tight and rough and may appear dull or flaky [4]. Unlike dry skin, which lacks oil, dehydrated skin lacks moisture and can affect all skin types, including oily, normal, dry, and combination skin [5]. Various strategies exist for managing skin health, such as maintaining proper hydration levels by using moisturizers. Moisturizers help preserve skin health by forming a natural barrier that retains moisture, thereby reducing signs of skin aging [1]. Effective regulation of skin hydration is vital for preserving skin health and developing new treatments for conditions like eczema [6,7], dry skin [1], and other dermatological issues related to hydration imbalance [8,9,10,11,12,13,14].

Several methods are developed to detect the skin hydration status, such as near infrared (NIR) multispectral imaging, which detects light absorption by water in skin tissues [15]. Other techniques include optical coherence tomography (OCT) [16], transient thermal transfer [17], nuclear magnetic resonance spectroscopy (NMR) [18], skin elasticity measurements [19], and electrochemical methods like skin thermodynamics and electrophoresis [20]. Detailed discussions of these methods can be found in [21]. Despite their benefits, these methods have significant drawbacks. OCT systems are expensive and require complex setups, making them impractical for routine use [22]. Transient thermal transfer can be uncomfortable and sensitive to ambient conditions, leading to inconsistent results [23,24]. NMR spectroscopy is costly, resource-intensive, and unsuitable for rapid or real-time analysis [18]. The impedance spectroscopy method has low accuracy because the applied pressure that is essential for electrical contact in these methods deforms the skin and thus affects its hydration status [10]. Furthermore, skin elasticity measurements often lack precision since they can be influenced by age and overall skin health [25]. Environmental factors can also affect skin thermodynamics, further complicating the measurement process. Electrophoresis is invasive, technically demanding, and requires specialized equipment, resulting in relatively low analytical accuracy [26,27,28]. On the other hand, commercially available corneometers are not wearable and are prone to errors due to applied pressure and angle of application during measurements [29]. These limitations necessitate a real-time, non-invasive, and cost-effective approach to measure skin hydration.

Microwave-based sensing methods have recently shown great potential in chemical, biological, and biomedical applications [30,31,32,33,34,35,36,37,38]. Microwave planar sensors offer intrinsic advantages such as low-cost fabrication, high sensitivity, real-time operation, and non-invasive detection, which are particularly attractive for biological and biomedical applications [39,40,41,42,43,44,45,46]. Given the distinct advantages of microwave technology, recent studies have reported significant efforts toward assessing skin lesions using microwave-based methods [47,48], demonstrating their strong potential for skin cancer diagnosis. Additionally, several sensors have been developed to detect skin hydration levels [27,49,50,51]. For example, the design in [49] uses a complementary split-ring resonator (CSRR) probe for hydration level detection in skin phantoms. However, such probes cannot be applied as wearable devices with real-time measurement capabilities. In [50], a planar split-ring resonator (SRR) coupled to a microstrip feed is proposed for real-time measurement of the skin hydration level. Although the sensor performs well in ex vivo measurements, adapting it for in vivo detection is challenging, as the skin must be sandwiched between the microstrip reader and the SRR during operation. A wearable microwave sensor, designed using a coplanar waveguide (CPW) on a flexible substrate, was recently reported in [27]. It shows strong potential for continuous in vivo monitoring of skin hydration in combination with a portable vector network analyzer (VNA). However, this sensor is relatively large, causing potential discomfort to the user (i.e., athlete) during regular activities. In addition, a reflective resonant microwave sensor was designed in [51] for in vivo detection of the tissue hydration level. Likewise, this sensor is relatively large, which hinders its adaptation for wearable purposes.

**Figure 1:**
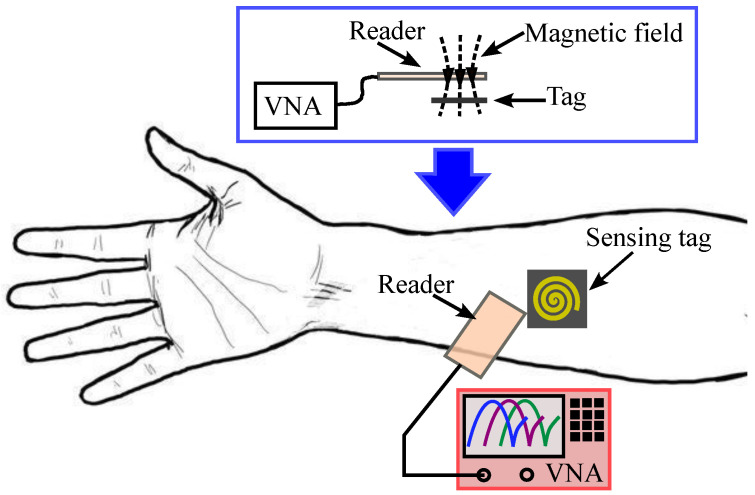
Conceptual operation principle of the proposed sensor.

This article presents the design of a new passive microwave sensor for in vivo monitoring of skin hydration. The sensor comprises a very compact resonant LC sensing tag that is wirelessly coupled to an inductive loop reader, as shown in Figure 1. Compared with the sensor in [50], the proposed design does not require the skin to be sandwiched between the sensing tag and the reader. Furthermore, unlike conventional inductively coupled tag sensors [52,53,54], the proposed sensor includes a 50Ω resistor in series with the inductive loop reader. Using this new design, the resonance frequencies can be directly detected using the $S_{11}$ magnitude without requiring any further processing to measure the input impedance. This significantly simplifies the measurement process compared with the existing sensor designs. The sensing tag is fabricated on a thin and flexible Teflon material (Rogers RT5880) covered with a thin Kapton film. It can be comfortably worn on the skin using a wristband or adhesive tape (Figure 1). The reader is inductively coupled to the tag and produces a resonant response with a reflection pole in the vicinity of a zero. The hydration level directly affects the skin dielectric constant, which, in turn, modifies the total capacitance of the sensing tag. This causes a shift in both the reflection zero and pole frequencies, which can be detected by measuring the reader’s reflection coefficient, as illustrated in the sensor’s conceptual framework in Figure 1. The sensing tag has a compact size (21×21 mm^2^), offering improved wearability and comfort compared to earlier designs.

In our study, we examine the skin treated with a moisturizer cream and Vaseline. The moisturizer (Dermal Therapy Very Dry Skin Cream, 28 g, Ego Pharmaceuticals, Heidelberg West, VIC, Australia) is designed to hydrate the skin and help retain moisture. It works by using a blend of ingredients that attract and retain water and may also provide additional benefits such as soothing or anti-aging effects. On the other hand, Vaseline (Vaseline® Petroleum Jelly, 100 g, Unilever Australia, North Rocks, NSW, Australia) acts by forming a barrier on the skin to prevent moisture loss. Unlike the moisturizer, it does not add moisture but helps retain the skin’s existing hydration by preventing evaporation.

This article is organized as follows: Section 2 explains the operating principles of the sensor and design methodology, along with the circuit model. Section 3 details the experimental setup and verification through measurements. Finally, Section 4 concludes the study.

## 2. Sensor Design and Circuit Analysis

**Figure 2:**
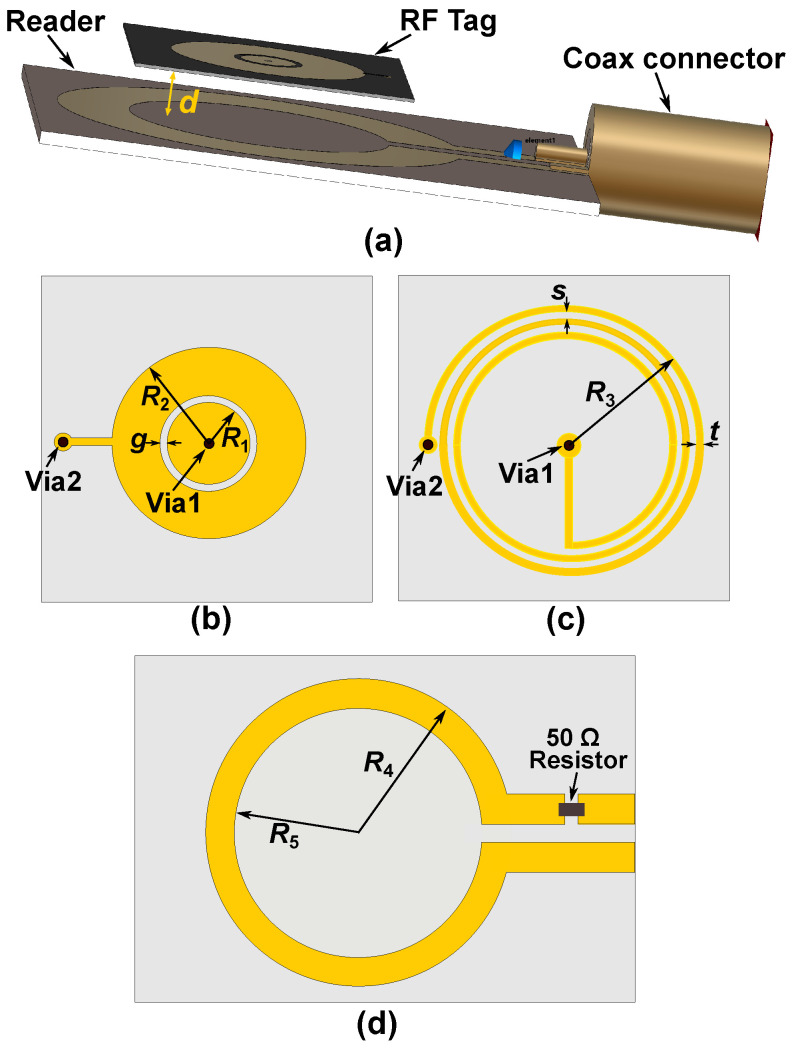
Schematic view of the designed sensor. (**a**) Prospective view of the wireless LC sensor for skin hydration measurement. (**b**) Sensing tag’s top view. (**c**) Sensing tag’s bottom view. (**d**) Top view of the reader coil. The geometrical dimensions are: $R_{1}=2.2$ mm, $R_{2}=7.7$ mm, g=0.5 mm, $R_{3}=9.2$ mm, t=0.3 mm, and s=0.3 mm, $R_{4}=10.3$ mm, $R_{5}=16$ mm.

The proposed wireless microwave sensor consists of two parts: a sensing tag and a reader. Configurations of the proposed radio frequency tag and the reader are shown in Figure 2. The figure shows the reader’s position relative to the tag, along with two-dimensional views of both the tag and the reader. The reader is made of an inductive loop in series with a 50Ω resistor. On the other hand, the sensing tag is made of a parallel LC resonator. The capacitance is designed on the top surface and is made of a circular metallic patch of radius $R_{1}$ enclosed by a metallic ring. An edge capacitance effect exists between the circular patch and the metallic ring along the gap *g*. The inductance is realized through a narrow spiral metallic path on the backside of the tag, as shown in Figure 2c. One end of the spiral inductor connects to the center of the circular patch via a metallic through-hole, while the other connects to the metallic ring through a second via, as shown in Figure 2b,c. This semi-lumped resonant structure comprising a spiral inductor and a planar capacitor results in a miniaturized resonator with dimensions significantly smaller than the wavelength at the operating frequency. The reader is a one-turn inductive metallic loop, as shown in Figure 2d, in series with a 50 Ω resistor. Inductive coupling is established by placing the reader and tag in close proximity. This allows detection of the resonance frequency variations caused by the skin hydration status. Such a physical geometry of the sensor offers several advantages over the other microwave-based skin hydration sensors. Some of the main advantages are as follows: First, the sensing tag’s flexible and compact structure allows it to be comfortably worn on the forearm, making it suitable for continuous real-time skin hydration monitoring. This feature is especially useful in managing conditions like eczema and in athletic settings. Second, the reader is separated from the main sensor, so only passive electronics are worn on the body. This reduces the size of the wearable component and enhances user comfort. Third, unlike conventional designs, the inductive and capacitive components are printed on opposite sides of the tag. This reduces the required area, resulting in a more compact sensor that improves user comfort. Furthermore, only the most sensitive part (the circular capacitive gap) contacts the skin.

**Figure 3:**
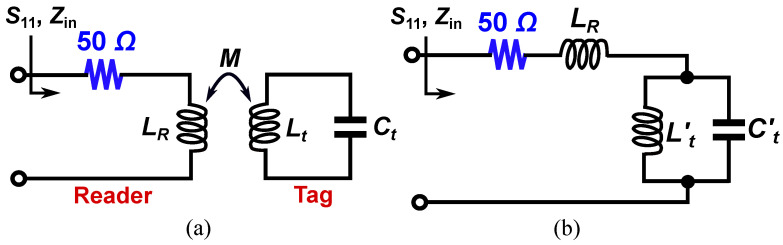
Lumped circuit model of the proposed sensor. (**a**) The reader tag and sensing tag are inductively coupled. (**b**) Simplified equivalent circuit model of the sensor.

An equivalent circuit model of the tag and the reader is presented in Figure 3a, where the inductive coupling is modeled with the mutual inductance *M*. In this model, the sensing tag is represented by an $L_{t}C_{t}$ resonant tank. Here, $C_{t}$ accounts for the edge capacitance between the circular disk and the surrounding metallic ring on the top side of the tag, while $L_{t}$ represents the inductance of the spiral coil located on the backside. In addition, $L_{R}$ is the equivalent inductance of the inductive loop reader that is in series with a lumped 50Ω resistor. This circuit model can be simplified to the one in Figure 3b. In the simplified circuit of Figure 3b, we have

(1) $$C_{t}^{\prime }=\frac{L_{t}}{\omega_{p}^{2}M^{2}}$$

(2) $$L_{t}^{\prime }=\omega_{p}^{2}M^{2}C_{t}$$

where

(3) $$\omega_{p}=2\pi f_{p}=\frac{1}{\sqrt{L_{t}C_{t}}}$$

is the resonance frequency of the tag.

According to the circuit model, at the resonance frequency of the tag defined by (3), the combination of $L_{t}^{\prime }$ and $C_{t}^{\prime }$ acts as an open circuit, resulting in the maximum reflection ($S_{11}=1$). Another important point is the resonance frequency of the composite resonator made of $L_{R}$, $L_{t}^{\prime }$, and $C_{t}^{\prime }$, which is defined as

(4) $$\omega_{z}=2\pi f_{z}=\frac{1}{\sqrt{\frac{L_{R}L_{t}^{\prime }}{L_{R}+L_{t}^{\prime }}C_{t}^{\prime }}}$$

At $f_{z}$, the combination of $L_{R}$, $L_{t}^{\prime }$, and $C_{t}^{\prime }$ acts as a short circuit, meaning that the input impedance $Z_{in}=50\;\Omega$. Thus, a perfect match happens at $\omega_{z}=2\pi f_{z}$ frequency, producing a notch in the reflection response at $f_{z}$. Such a resonance can be easily detected just by measuring $|S_{11}|$. By comparing Equations (3) and (4), it is clear that the reflection zero frequency ($f_{z}$) is larger than $f_{p}$, where the maximum reflection occurs.

In the sensor design, the geometrical dimensions of the tag and the reader are designed based on the required tag size to produce a good comfort to the user and the operational frequency. The operational frequency range is selected around 50–200 MHz since according to [55,56], human skin shows a large variation in the permittivity value as a function of hydration. Then, the maximum dimension of the tag ($R_{3}$) is selected to be 9.2 mm since it causes a relatively compact structure, offering a good comfort to the user when wearing the tag sensor. Next, $R_{5}$ is selected to be larger than $R_{3}$ to produce a good inductive coupling between the reader and the tag when the tag is placed inside the area of the reader loop for measurements. The capacitive gap (*g*) has been selected to be 0.5 mm, offering a good penetration of the E-field from the tag into the skin for hydration measurements [40]. The other geometrical dimensions are well tuned using the full-wave simulations in the CST Microwave Studio to bring the operation frequency to around 50–200 MHz when performing skin hydration measurements.

**Figure 4:**
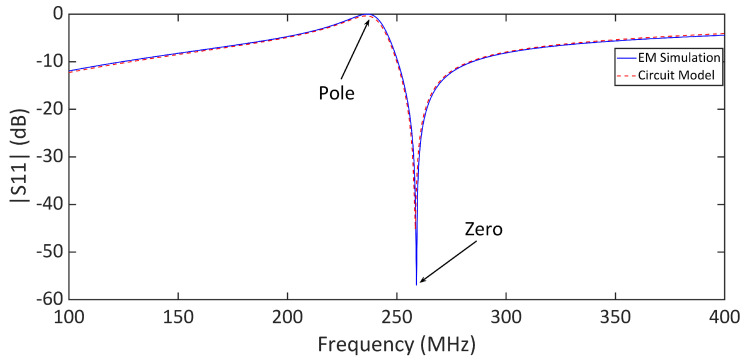
Comparison between the full-wave electromagnetic (EM) and circuit model simulation results of the sensor with a bare tag. The distance between the reader and tag is 0.1 mm. The element values of the circuit model are R=50Ω, $L_{R}$ = 33.5 nH, ${L^{\prime }}_{t}$ = 6.66 nH, and ${C^{\prime }}_{t}$ = 68 pF.

**Figure 5:**
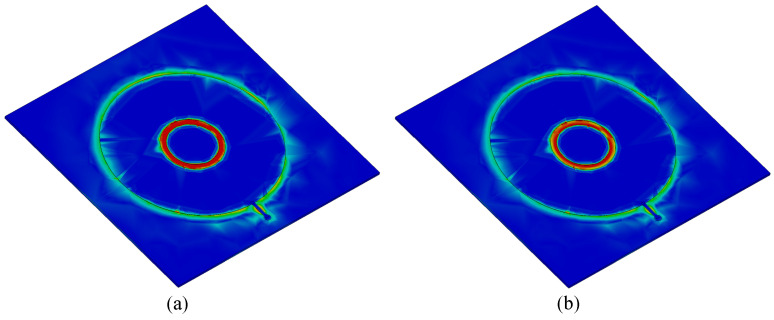
Full-wave simulated electric field (E-field) distribution at the transmission pole and zero frequencies of the bare sensor. (**a**) E-field at pole frequency. (**b**) E-field at zero frequency.

Full-wave electromagnetic (EM) simulations were carried out in CST Microwave Studio, where the complete reader–tag structure was modeled, as shown in Figure 2a. Figure 4 presents a comparison between the full-wave electromagnetic (EM) and the circuit model simulation results of a typical sensor, where the distance between the reader and the bare tag is 0.1 mm. The substrate used in the design of the LC tag is a 0.127 mm thick Rogers RT5880 with $\varepsilon_{r}=2.2$ and tanδ=0.0009, whereas the reader is designed on a 0.8 mm thick FR4 substrate with $\varepsilon_{r}=4.6$ and tanδ=0.025. The geometrical dimensions of the tag and reader are given in the caption of Figure 2. The strong agreement between the results in Figure 4 validates the presented circuit model analysis. The extracted values of the circuit elements are given in the caption of Figure 4. Figure 5 illustrates the electric field (E-field) distribution at the pole and zero frequencies. The highest electric field intensity is observed in the capacitive area, representing the sensor’s main sensitive part to any dielectric loading.

**Figure 6:**
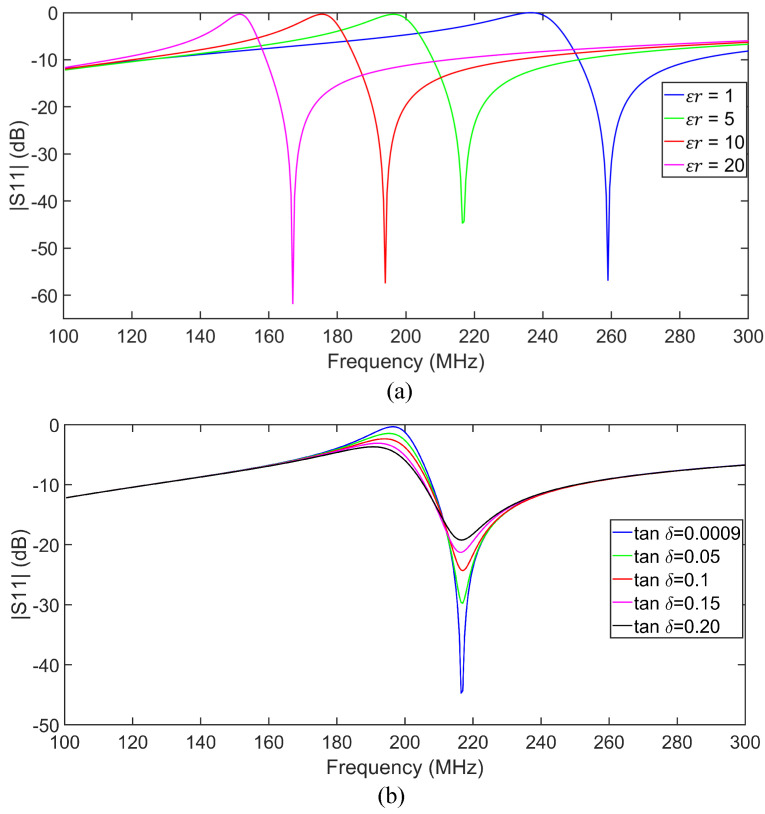
(**a**) Full-wave simulated $|S_{11}|$ results for various permittivity values of a cylindrical dielectric slab loaded on the sensing gap of the tag. (**b**) Full-wave simulated $|S_{11}|$ for a dielectric sample with $\varepsilon_{r}=5$ and various loss tangent values.

The sensor response was also investigated under various dielectric loadings, including air ($\varepsilon_{r}=1$) and four additional samples with relative permittivities of $\varepsilon_{r}=5,10,15$, and 20. In these simulations, we kept the loss tangent of the samples very small (tanδ=0.0009) to just study the effect of sample permittivity on the sensor response. In simulations, 1 mm thick dielectric samples were placed on top of the circular capacitive patch of the tag covering the whole sensitive area (see Figure 5). Figure 6a shows that both pole and zero resonance frequencies shift down as $\varepsilon_{r}$ increases. To evaluate the impact of loss tangent on sensor performance, additional simulations were performed at $\varepsilon_{r}=5$, with the loss tangent varying from 0.0009 to 0.2. The results are shown in Figure 6b. As seen, increasing the loss tangent primarily affects the return loss levels at the pole and zero frequencies. The skin hydration level significantly affects the complex permittivity of the skin, as proven in [27,49,50,51]. Thus, an LC resonant tag can detect variations in skin hydration levels based on the resonance frequency variation if the tag is in contact with the skin. Both the pole ($f_{p}$) and zero ($f_{z}$) frequencies can be used for sensing as shown in Figure 6. Note that according to (3) and (4), $f_{z}>f_{p}$ always, ensuring no frequency overlap.

## 3. Experimental Setup and Measurements

**Figure 7:**
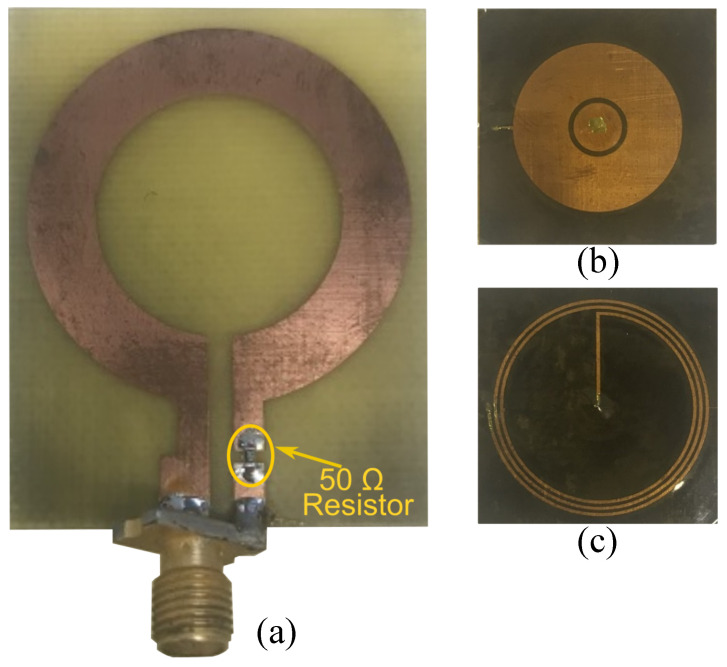
Fabricated reader and tag. (**a**) Top view of the reader coil, (**b**) top view of the sensing tag, and (**c**) bottom view of the sensing tag.

To validate the skin hydration sensing concept, the sensing tag and reader coils were fabricated on the substrates described in Section 2. The sensing tag and the reader are fabricated using a precision printed circuit board (PCB) laser milling machine offering a high precision in realizing small feature sizes. The geometrical dimensions used for fabrication are listed in the caption of Figure 2. The side dimensions of the reader’s substrate are 36mm×44mm, while the sensor tag measures 21mm×21mm. Photographs of the fabricated tag and the reader are presented in Figure 7. A lumped 50Ω resistor is soldered in series with the inductive loop reader. An SMA port is soldered to the reader for the measurements.

### 3.1. Experiment Setup

**Figure 8:**
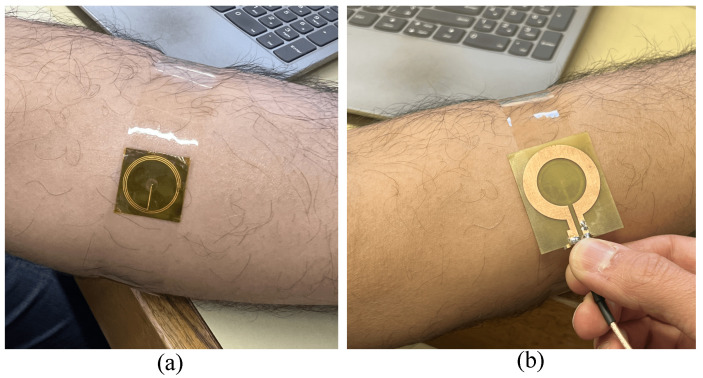
Experimental setup. (**a**) Sensor tag placed on the forearm. (**b**) Reader coil placed on sensor tag.

Figure 7 shows the experimental setup, where the sensor was affixed to the subject’s forearm using adhesive tape to ensure stable contact and consistent readings. During measurements, the subject’s hand was rested on a table to minimize unintended movement. In the experiments, the sensitive part of the tag (top side with the circular capacitive patch) was covered with a 0.04 mm thick Kapton tape, as shown in Figure 7c. Subsequently, the reader was positioned over the tag to capture the reflection responses. As shown in Figure 8b, the back side of the reader substrate is in touch with the back side of the tag (resembling a 0.8 mm distance between the reader metallization and the tag) in all of the measurements. The objective was to evaluate the performance of the wireless sensing system for detecting skin hydration changes by analyzing the reflection coefficient ($S_{11}$) over the 50–200 MHz frequency range. Before the measurements, the VNA was calibrated with open, short, and load standards within the 50–200 MHz frequency range. The reader was then connected to the VNA via an extension cable to perform the measurements.

The experiment assesses skin hydration under four conditions: wet skin, dry skin, skin treated with moisturizer lotion, and Vaseline-treated skin. First, the measurements were performed on the dry skin. Then, for the wet condition, a wet tissue was placed on the subject’s forearm for 30 min, with additional water applied to maintain consistent hydration. Excess water was carefully removed before the first measurement. Following this, the subject remained at room temperature for another 30 min to allow the skin to return to its normal state. Next, moisturizer lotion was applied, and after ensuring proper absorption, a new set of measurements was taken. The skin was then thoroughly cleaned with ethanol and tissue to remove any residue before the final application of Vaseline, followed by the last measurement set. The measurements were repeated five times for each state of the skin to ensure consistency.

All measurements were conducted throughout the experiment at a controlled room temperature of 23 °C. The sensor was placed consistently on the forearm, with a reference circle drawn around the placement area to ensure precision across all conditions. The sensor was cleaned with ethanol before each measurement set to avoid errors caused by surface contamination. During data collection, the subject’s arm rested on a table to minimize movement. The reader coil positioned near the sensor ensured optimal signal transmission. Once a stable setup was achieved, $S_{11}$ measurements were recorded under various skin hydration states.

### 3.2. Measured Results

**Figure 9:**
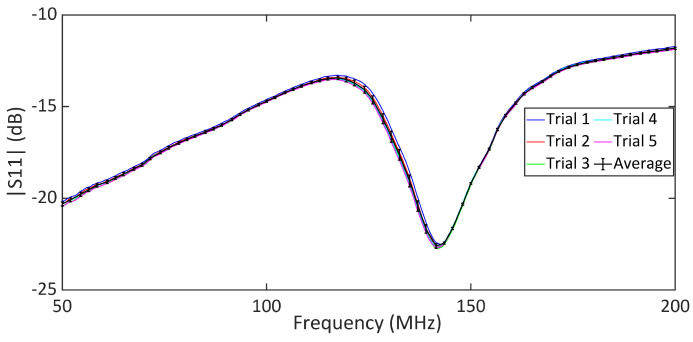
Measured $|S_{11}|$ for dry skin, where five iterative measurements are presented.

**Figure 10:**
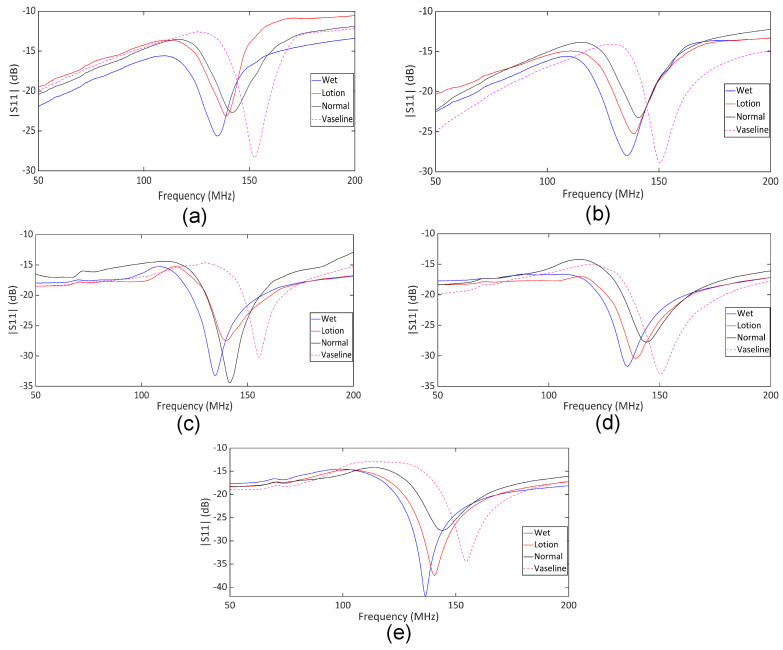
Measured $|S_{11}|$ under four conditions: wet skin, dry skin (normal), skin treated with lotion, and skin covered with Vaseline. (**a**) Subject 1, (**b**) Subject 2, (**c**) Subject 3, (**d**) Subject 4, and (**e**) Subject 5.

The comparative analysis of the measured $|S_{11}|$ data for different skin conditions provides further insights into the sensor’s performance in differentiating between various hydration levels. To this end, we performed experimental measurements on five human subjects according to the described measurement procedure. Ethical approval for this study was granted by the Department of Biomedical Engineering, RMIT University. Each subject participated in a one-hour session involving the application of moisturizer lotion, distilled water, and Vaseline to the forearm, as outlined in the measurement protocol. Figure 9 shows the measured $|S_{11}|$ response for untreated dry skin across five repeated trials. These data show that the poles and zeros remained unchanged across the trials, indicating that our measurements were stable and reliable for normal skin hydration conditions. The measured return loss is high due to the lossy nature of the human skin. However, the reflection can be easily detected using a benchtop VNA, as shown through the measurements. The same measurement can also be performed using low-cost portable VNAs, enabling a compact setup suitable for wearable applications. The plots in Figure 10a–e show the in vivo measured responses of the sensor under different skin hydration levels across four conditions for five human subjects. The measured results for all subjects are presented under four conditions: wet skin, skin covered with moisturizer lotion, dry skin (normal), and skin covered with Vaseline. The measurement of each skin hydration state was repeated five times for each subject, resulting in 100 measured data sets. Figure 10 shows that across all subjects, both the transmission pole and zero frequencies exhibit a downward shift from the dry to the fully hydrated condition. Such a frequency shift is attributed to the variation in the skin dielectric properties under different hydration states, which significantly affect the sensor’s overall capacitance. Water, with a high relative permittivity ($\varepsilon_{r}=80$), plays a critical role in the sensor’s sensitivity. In fact, water absorption in skin causes notable variations in the sensor’s overall capacitance, allowing it to effectively monitor hydration levels. Dry human skin typically exhibits a dielectric constant ranging from 30 to 60 within the measurement frequency range, depending on its hydration level [55,56]. This variation in dielectric properties enables our sensor to detect subtle changes in skin hydration, making it a powerful tool for real-time assessments. On the other hand, Vaseline® Petroleum Jelly has a much lower dielectric constant of around 2 [57]. Thus, applying Vaseline decreases the overall dielectric constant of the skin and causes an upward shift in the sensor’s response. As shown in Figure 10, the pole and zero frequencies across all subjects exhibit distinct shifts under different hydration conditions.

**Figure 11:**
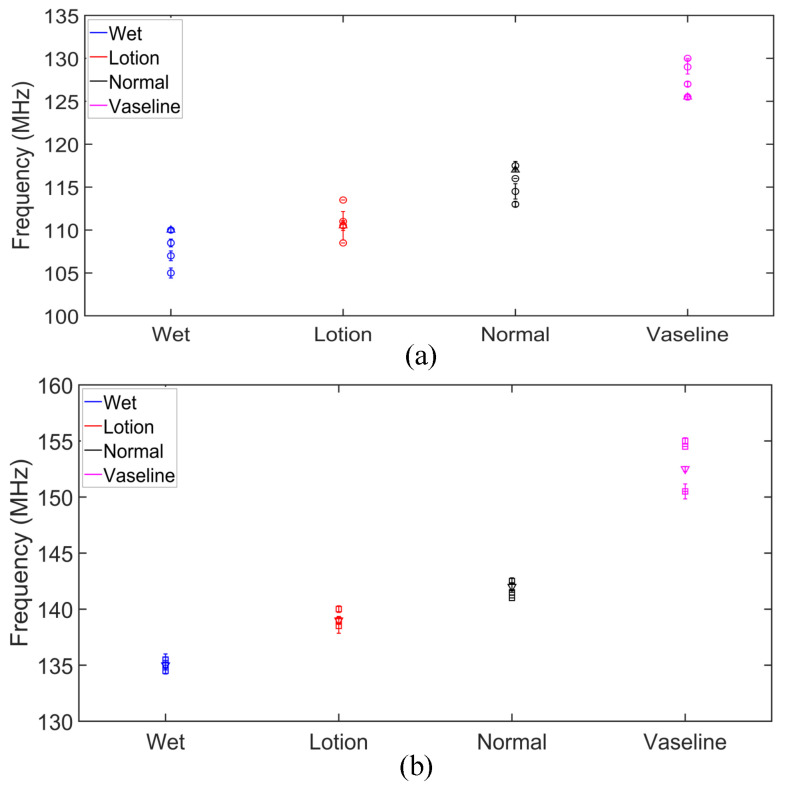
Measured pole and zero frequencies of five subjects under different skin conditions. (**a**) Pole frequencies of Subject 1–5. (**b**) Zero frequencies of Subject 1–5.

**Figure 12:**
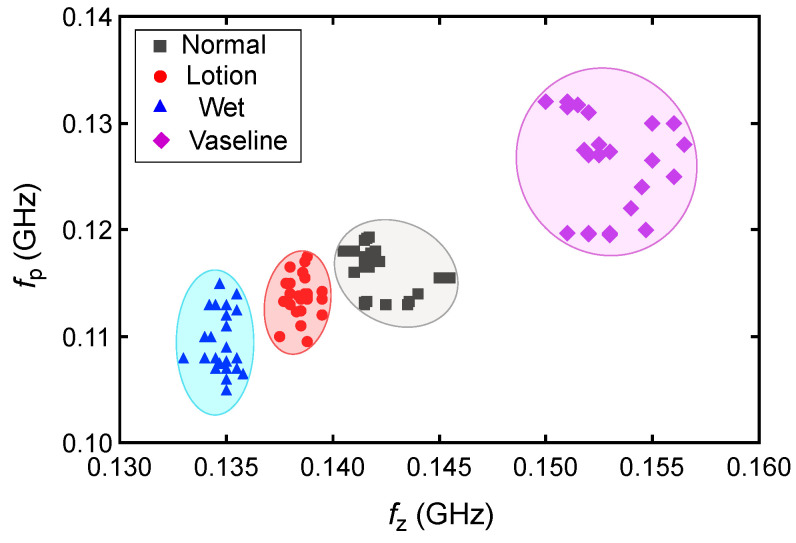
Measured $f_{p}$ and $f_{z}$ frequencies for all of the 100 measurements performed on five human subjects.

The measured transmission zero and pole frequencies of the five human subjects are presented in Table 1 and plotted in Figure 11 for different skin conditions. We presented the data with standard deviations (SD) from five measurements. This helps with a better understanding of the measurement consistency and precision by quantifying the observed small variations. The results in Figure 11 show a good agreement between the measured pole and zero frequencies of the five subjects under different skin hydration conditions. The slight fluctuations in the $f_{z}$ and $f_{p}$ frequencies in different measurements can be explained through different skin textures and properties among different subjects, as well as slight differences between the hydration levels and/or the amount of applied lotion and Vaseline in different repetitions of the tests. Based on the results in Figure 10, the sensor response in different hydration states might vary a little bit among individual subjects, mainly due to slight differences between the skin properties among individuals. Thus, calibrations would be required for individual users of the sensor. To this end, we repeated measurement of each state five times for each test subject and plotted the corresponding $f_{z}$ and $f_{p}$ for a hundred measurements in Figure 12. As seen, specific regions can be identified for different hydration states. The four different skin conditions can be clearly distinguished, confirming the proposed sensor’s effectiveness in detecting skin hydration states. For example, the normal skin condition is identified as $140.5<f_{z}<145.33$ MHz and $113<f_{p}<119$ MHz. According to the results in Table 1, for subject 1, the maximum standard deviation for the pole frequency was ±1.66 MHz under the lotion-applied condition, while the maximum zero frequency standard deviation was ±0.42 MHz for multiple conditions. Similarly, for subject 2, the maximum pole frequency standard deviation was ±0.89 MHz under dry skin condition, and the maximum zero frequency standard deviation was ±0.67 MHz under the Vaseline-applied condition. For subject 3, the maximum pole frequency standard deviation was ±0.57 MHz under the wet skin condition, while the maximum zero frequency standard deviation was ±0.29 MHz under the wet skin conditions. For subject 4, the maximum pole frequency standard deviation was ±0.5 MHz under the normal skin (untreated) condition, and the maximum zero frequency standard deviation was ±0.29 MHz under the lotion-applied conditions. For subject 5, the maximum pole frequency standard deviation was ±0.58 MHz under the wet skin condition, while the maximum zero frequency standard deviation was ±0.5 MHz for the wet skin conditions. The maximum standard deviation (SD) of the measured $f_{z}$ is 0.5 MHz for $f_{z}=135.5$ MHz, which happens for subject 5 under the wet condition. This means less than 0.037% error. The maximum SD of the measured fp is 1.66 MHz, which happens for subject 1 under the lotion applied skin condition. This translates to less than 1.5% error. Such a performance indicates the consistency of the proposed sensor response while used for skin hydration monitoring for individual human subjects. This capability is especially valuable in applications such as skincare, dermatology, and sport, where accurate and timely assessments of skin hydration are essential. The slight discrepancy in the measured pole and zero frequencies of the subjects are attributed to the differences in skin properties and moisture levels of distinct individuals. Despite these variations, the poles and zeros for both subjects were closely aligned, confirming that the sensor can provide accurate and repeatable measurements across different individuals. Similar to any other kind of sensors, there are limitations associated with the proposed sensing method. For example, since the measurement is performed based on dielectric properties of the skin, sweating or applying cosmetics, creams might interfere with the sensor measurement as these factors might affect the dielectric properties of the skin. Other factors that might limit the accuracy of the sensor through affecting the skin dielectric properties are the blood sugar level, electrolyte level, and body temperature.

### 3.3. Future Work and Development

For future potential development of a full wearable and real-time hydration monitoring, a dedicated wristband can be designed to hold the reader securely over the sensing tag, preventing its detachment or displacement from the forearm due to body movements. In addition, a portable VNA or a dedicated electronic readout circuit should be developed for that purpose. On the other hand, because the sensing tag is flexible, body movement that causes excessive bending can deform the sensing capacitor and reduce measurement accuracy. Therefore, it is recommended to apply the sensing patch to a flat area of the body such as the forearm, as used in our tests. The shift in $f_{z}$ and $f_{p}$ can be used for quantitative hydration level monitoring, but this requires calibration against a gold standard method (i.e., body mass change, urine tests, saliva, rating of thirst, or isotope dilution techniques). This necessitates huge data analysis and measurement for each individual test subject. This would be a subject of future work for further development of the sensor for a quantitative measurement of the skin hydration level.

In summary, the data from all the subjects illustrate that our sensor can detect different skin hydration statuses. Our microwave sensor stands out from conventional methods due to its real-time, non-invasive, and cost-effective approach to measuring skin hydration. Unlike traditional techniques, such as OCT, which is expensive and complex, or NMR spectroscopy, which is costly and not suitable for rapid analysis, our sensor offers real-time, non-invasive, and accurate hydration detection without discomfort or complex setup. It directly links changes in the skin’s dielectric permittivity to hydration levels, offering a straightforward and precise alternative. Our microwave sensor is suitable for continuous and practical skin hydration monitoring. Although the measurements are taken using a bench top VNA, in reality, the measurement can also be performed using low-cost portable VNAs offering a compact setup suitable for portable and wearable applications. This is evidenced by the shifts in resonance frequency between normal, wet, and lotion-applied skin conditions observed in our study. These shifts align with findings from other research on microwave sensors used for bio-sensing applications, where the ability to distinguish between different hydration states has been recognized as a critical feature [27,50,58]. A comparison between the proposed sensors and the other microwave-based hydration sensors is presented in Table 2. According to the table, the designed sensor has a very compact size with flexible structure offering the most comfort among the microwave wearable solutions for skin hydration monitoring.

## 4. Conclusions

In this study, we developed and thoroughly evaluated a microwave sensor designed for real-time skin hydration monitoring. The sensor features a semi-lumped LC resonator on a flexible microwave substrate. The sensor differentiates skin hydration states, including wet, moisturizer applied, normal, and Vaseline-treated skin according to the variations in the skin dielectric properties. The shifts in resonance frequency observed in the $S_{11}$ measurements provided clear evidence of the sensor’s sensitivity to changes in hydration levels, confirming its potential as a reliable tool for skin hydration assessment. The sensor’s operation principle was analyzed and explained through an equivalent circuit model that accurately predicts the reflection zero and pole frequencies. The full-wave simulations and measured results verified the circuit model, further solidifying the presented analysis.

Testing the sensor on five subjects revealed distinct pole and zero frequencies for each skin condition, validating the sensor potential in recognizing different hydration states of the skin. The low standard deviations observed across the measurements indicate that the sensor provided consistent and reliable results. Overall, our study highlights the potential of the proposed sensor in tracking hydration changes accurately and in real-time. Its ability to consistently differentiate between various skin hydration states makes it a promising tool for applications in skin health monitoring and broader biomedical fields. 

## Figures and Tables

**Table 1:** Analysis of $f_{p}$ and $f_{z}$ Frequencies and Standard Deviation (SD) for Different Hydration States for Subjects 1 to 5.

Subj.	Conditions	Pole Freq. $f_{p}$ (MHz)	Pole SD (±MHz)	Zero Freq. $f_{z}$ (MHz)	Zero SD (±MHz)
1	Wet	110.0	0.27	135.0	0.42
	Lotion	110.5	1.66	139.0	0.35
	Normal	117.0	0.22	142.0	0.35
	Vaseline	125.5	0.35	152.5	0.22
2	Wet	108.5	0.45	135.5	0.22
	Lotion	110.5	0.55	138.5	0.65
	Normal	114.5	0.89	141.0	0.00
	Vaseline	129.0	0.82	150.5	0.67
3	Wet	107.0	0.57	134.5	0.29
	Lotion	111.0	0.10	139.0	0.28
	Normal	116.0	0.00	141.5	0.00
	Vaseline	130.0	0.00	155.0	0.28
4	Wet	110.0	0.29	135.0	0.00
	Lotion	113.5	0.00	139.0	0.29
	Normal	117.5	0.50	141.5	0.00
	Vaseline	125.5	0.00	150.5	0.00
5	Wet	105.0	0.58	135.5	0.50
	Lotion	108.5	0.29	140.0	0.29
	Normal	113.0	0.29	142.5	0.29
	Vaseline	127.0	0.29	154.5	0.00

**Table 2:** Comparison with previous microwave skin hydration sensors.

Ref.	Structure	$f_{0}$ (GHz)	Wearable Potential	Size (${\mathrm{mm}}^{2}$)
[27]	Flexible CPW	0.35	Yes	50×90
[49]	CSRR probe	5.5	No	6.4×6.4
[50]	SRR tag	1	No	56.8×54.6
[51]	CPW Res.	0.9	No	34.6×34.6
This Work	Flexible LC tag	0.13	Yes	21×21

## Data Availability

There is no data associated with this paper.
